# Supplementary material for: Influenza A virus undergoes compartmentalized replication in vivo dominated by stochastic bottlenecks
Source: Nat Commun. 2022 Jun 14;13:3416. doi: 10.1038/s41467-022-31147-0 (PMC9197827; doi:10.1038/s41467-022-31147-0)
Supplement: Supplementary file 1 — Supplementary Information [file 41467_2022_31147_MOESM1_ESM.pdf]

Supplementary Information for Amato, et al.

Contents:

Supplementary Figure 1. Replicate sequencing of viral stock with an NheI registration mark highlights reproducibility of barcode enumeration. Associated with Figure 3.

Supplementary Figure 2. Replicate sequencing of viral stock with a PstI registration mark highlights reproducibility of barcode enumeration. Associated with Figure 3.

Supplementary Figure 3. Diverse populations in mouse lungs are partially correlated with high frequency members in the inoculum at early times during infection. Associated with Figure 4.

Supplementary Figure 4. Viral titers from trachea and lung and replication of reduced populations in the trachea. Associated with Figure 5.

Supplementary Figure 5. Forward model simulations of lineage dynamics in ferret lungs. Associated with Figure 6.

Supplementary Figure 6. Bottlenecks result in stochastic outgrowth of viruses in lung lobes. Associated with Figures 6-7.

Supplementary Figure 7. Tracking barcodes throughout the respiratory tract. Associated with Figures 5-7.

Supplementary Table 1. List of primers used for design and deep sequencing of barcoded-H1N1 virus

Supplementary Table 2. List of indexes used for deep sequencing of barcoded-H1N1 virus

Supplementary Table 3: Accession numbers for sequencing data

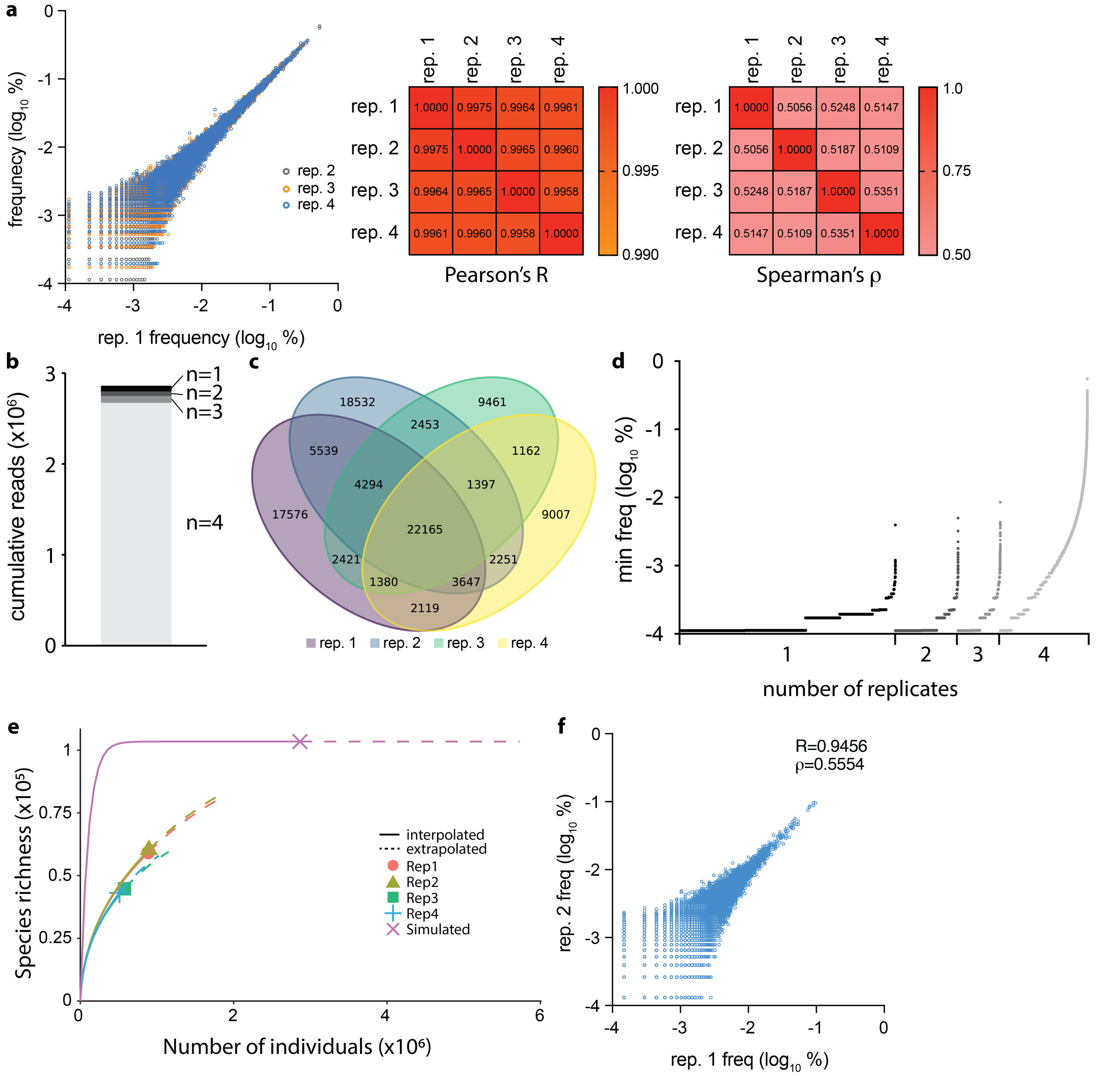

Supplementary Figure 1. Replicate sequencing of viral stock with an NheI registration mark highlights reproducibility of barcode enumeration. Associated with Figure 3. a) The barcodes present on HA in virus stock with the NheI registration mark were subject to 4 replicate sequencing runs to assess reproducibility. The frequency of individual barcodes in replicates 2-4 are plotted relative to their frequency in replicate 1 (left). Pearson's R and Spearman's ρ correlation coefficients calculated between all replicate pairs (right). b) Almost all HA barcode sequencing reads are shared in all four replicates. Sequence reads were grouped based on their presence in n = 1, 2, 3 or 4 replicates. c) Venn diagram highlighting overlap of HA barcode identity in replicate sequencing, independent of the abundance of any individual barcode. d) Replicate sequencing of HA barcodes reproducibly enumerates all but the lowest frequency barcodes. Barcodes were separated based on their presence in 1, 2, 3 or 4 replicates. The minimum frequency of an individual barcode across all replicates was plotted. Barcodes with the lowest frequency tend to appear in only a subset of replicates. e) Rarefaction-extrapolation curves of replicate sequencing runs compared to simulated data generated by Poisson sampling of a hypothetically ideal population where all barcodes are equally represented. f) Replicate sequencing of PA barcodes present in virus stock with the NheI registration mark. R = Pearson's correlation coefficient. ρ = Spearman's rank correlation coefficient.

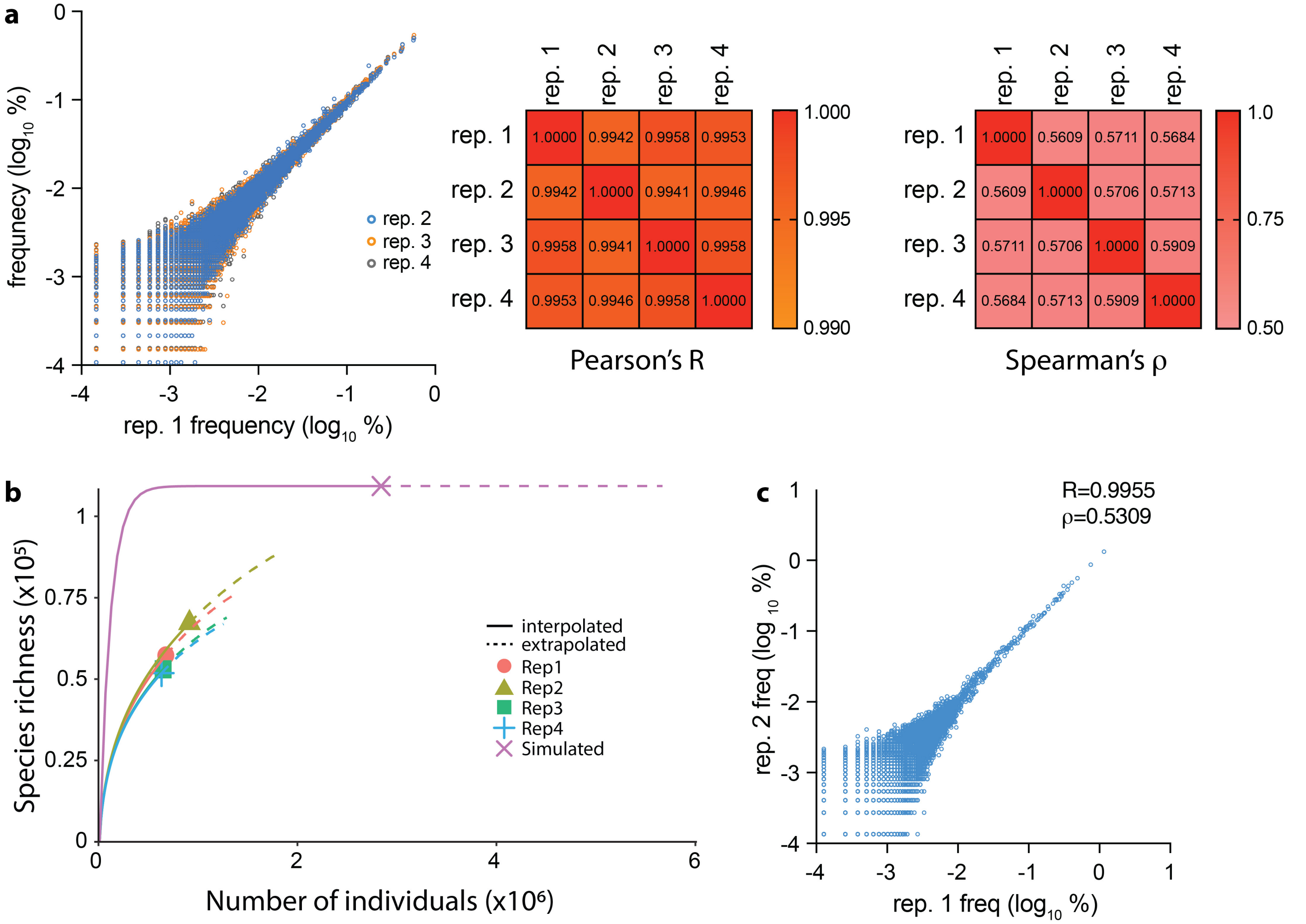

Supplementary Figure 2. Replicate sequencing of viral stock with a PstI registration mark highlights reproducibility of barcode enumeration. Associated with Figure 3. a) The barcodes present on HA in virus stock with the PstI registration mark were subject to 4 replicate sequencing runs to assess reproducibility. The frequency of individual barcodes in replicates 2-4 are plotted relative to their frequency in replicate 1 (left). Pearson's R and Spearman's ρ correlation coefficients calculated between all replicate pairs (right). b) Rarefaction-extrapolation curves of replicate sequencing runs compared to simulated data generated by Poisson sampling of a hypothetically ideal population where all barcodes are equally represented. c) Replicate sequencing of PA barcodes present in virus stock with the PstI registration mark. R = Pearson's correlation coefficient. ρ = Spearman's rank correlation coefficient.

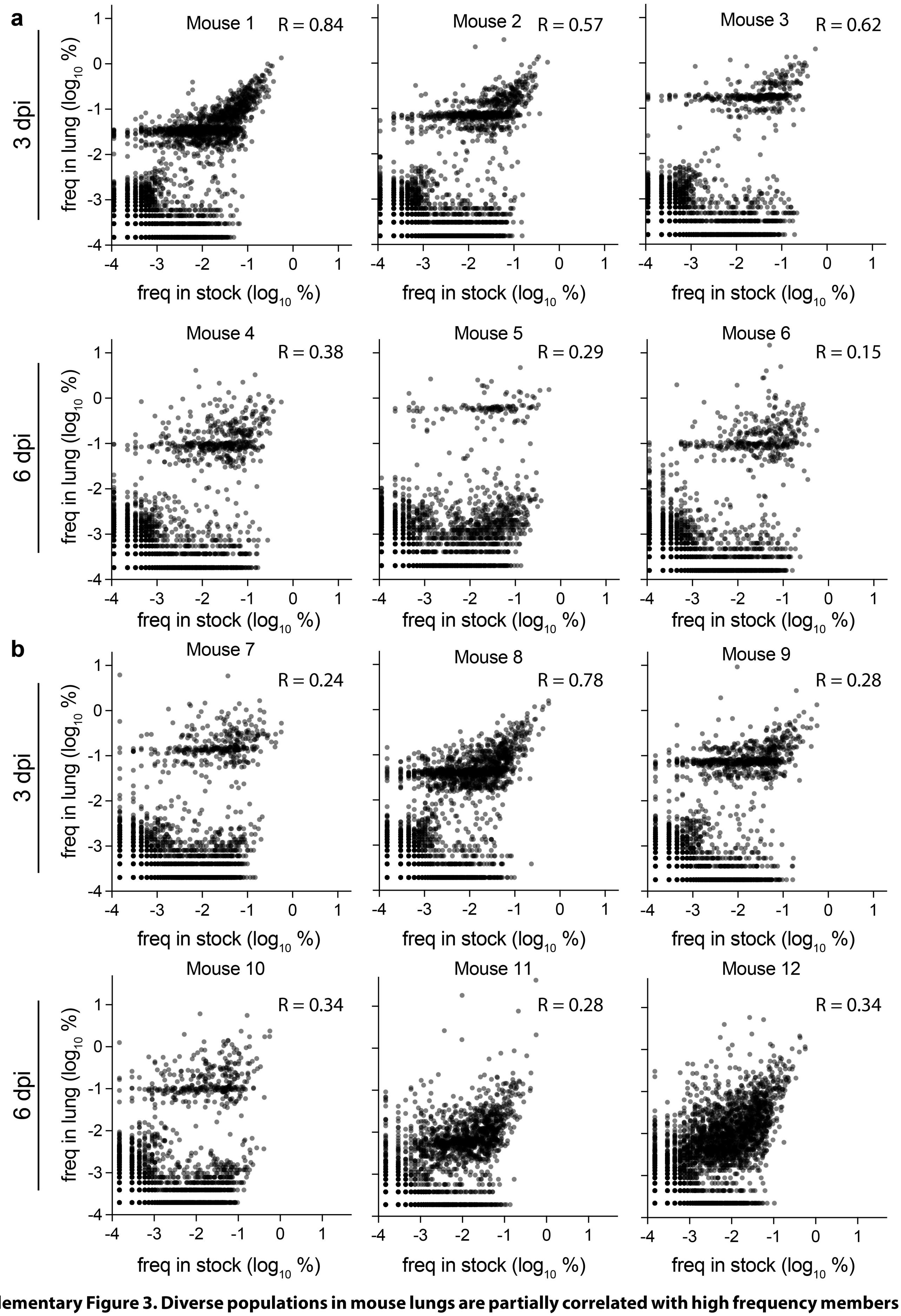

Supplementary Figure 3. Diverse populations in mouse lungs are partially correlated with high frequency members in the inoculum at early times during infection. Associated with Figure 4. A) The frequency of lineages in the inoculum was compared to those in mice inoculated with HA-K153E-NheI libraries at 3 and 6 dpi. B) Same as A for the HA-K153E-PstI libraries. R = Pearson's correlation coefficient.

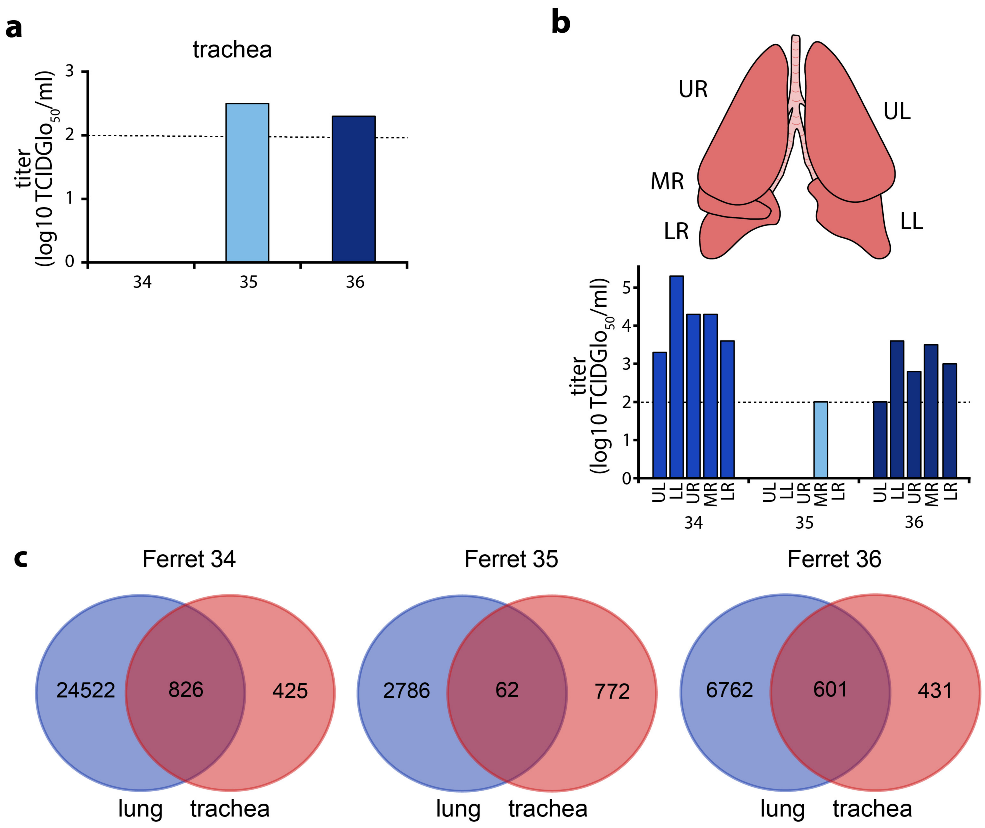

Supplementary Figure 4. Viral titers from trachea and lung and replication of reduced populations in the trachea. Associated with Figure 5. a) Virus was recovered from the trachea of infected ferrets at 5 dpi and viral titers were measured by $TCIDGlo_{50}$. b) Viral populations in individual lung lobes were titered by $TCIDGlo_{50}$. c) Venn diagram of lineages present in the trachea and all lung lobes at 5 dpi. The limit of detection for viral titer assays is indicated by a dashed line in A and B.

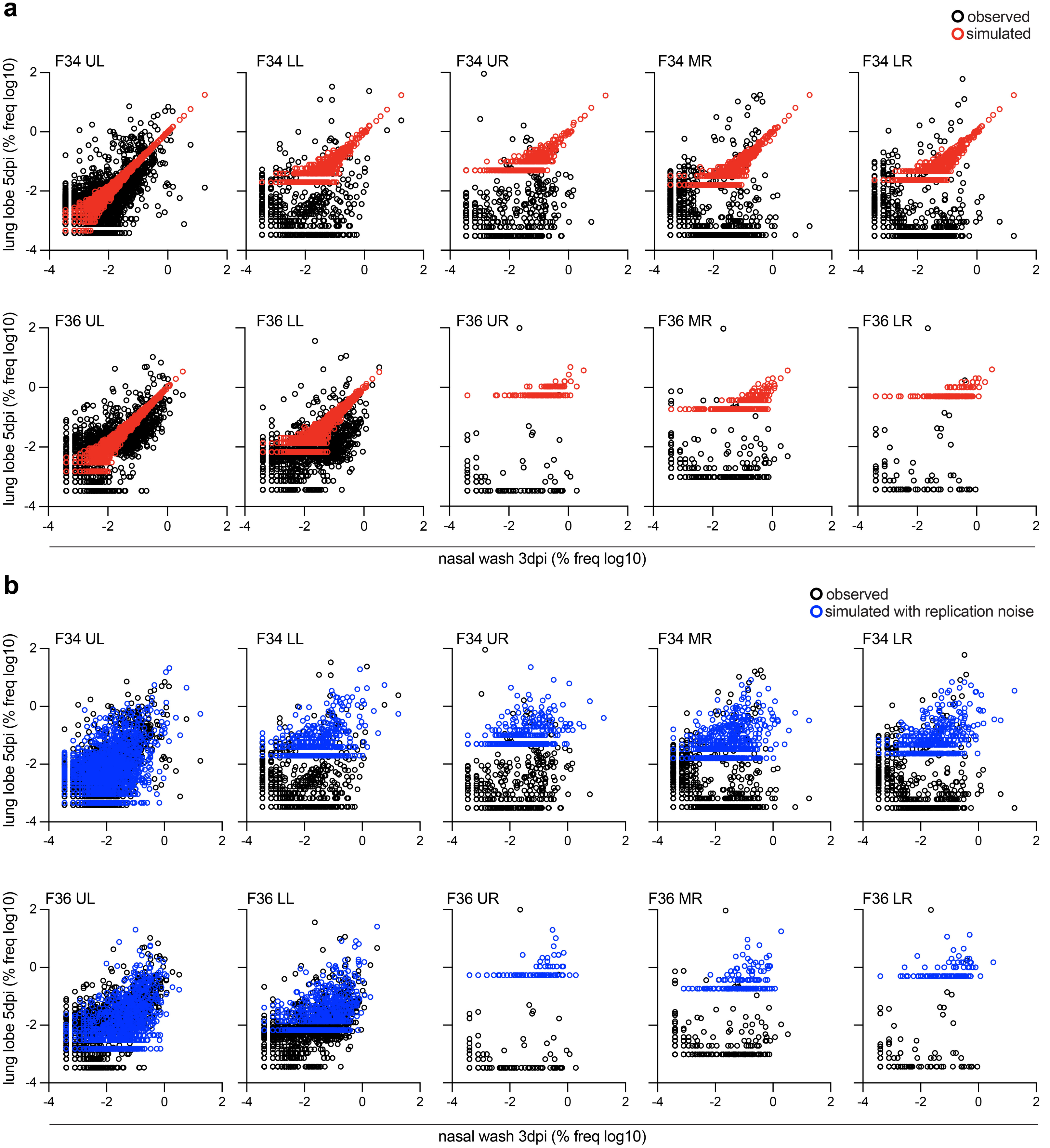

Supplementary Figure 5. Forward model simulations of lineage dynamics in ferret lungs. Associated with Figure 6. a) Forward model simulations with wide bottlenecks provide predictions of higher lineage frequency similarity than those observed. Mock datasets were simulated, where the starting population in nasal washes from a ferret at 3 dpi passes through a bottleneck of size N_b, yielding predicted lineage frequencies in each of the lung lobes. N_b values in the forward simulations were set to their maximum likelihood estimates shown in Figure 6D. Modeled data (red) show a strong correlation between frequency in the donor and recipient populations, whereas observed frequencies (black) show large differences in frequencies. The top row shows results for ferret 34 and the bottom row shows results for ferret 36. Simulated frequencies are bounded from below at frequencies of $1/N_b$ because viral growth is not explicitly modeled. b) Forward model simulations with wide bottlenecks together with stochastic local replication dynamics generate lineage frequency patterns similar to those observed. Mock datasets were simulated, where the starting population in nasal washes from a ferret at 3 dpi passes through a bottleneck of size N_b, yielding predicted lineage frequencies in each of the lung lobes. Model predictions included the process of environmental noise. N_b values in the forward simulations were set to their maximum likelihood estimates shown in Figure 6D. Modeled data (blue) better reflect observed frequencies (black). The top row shows results for ferret 34 and the bottom row shows results for ferret 36.

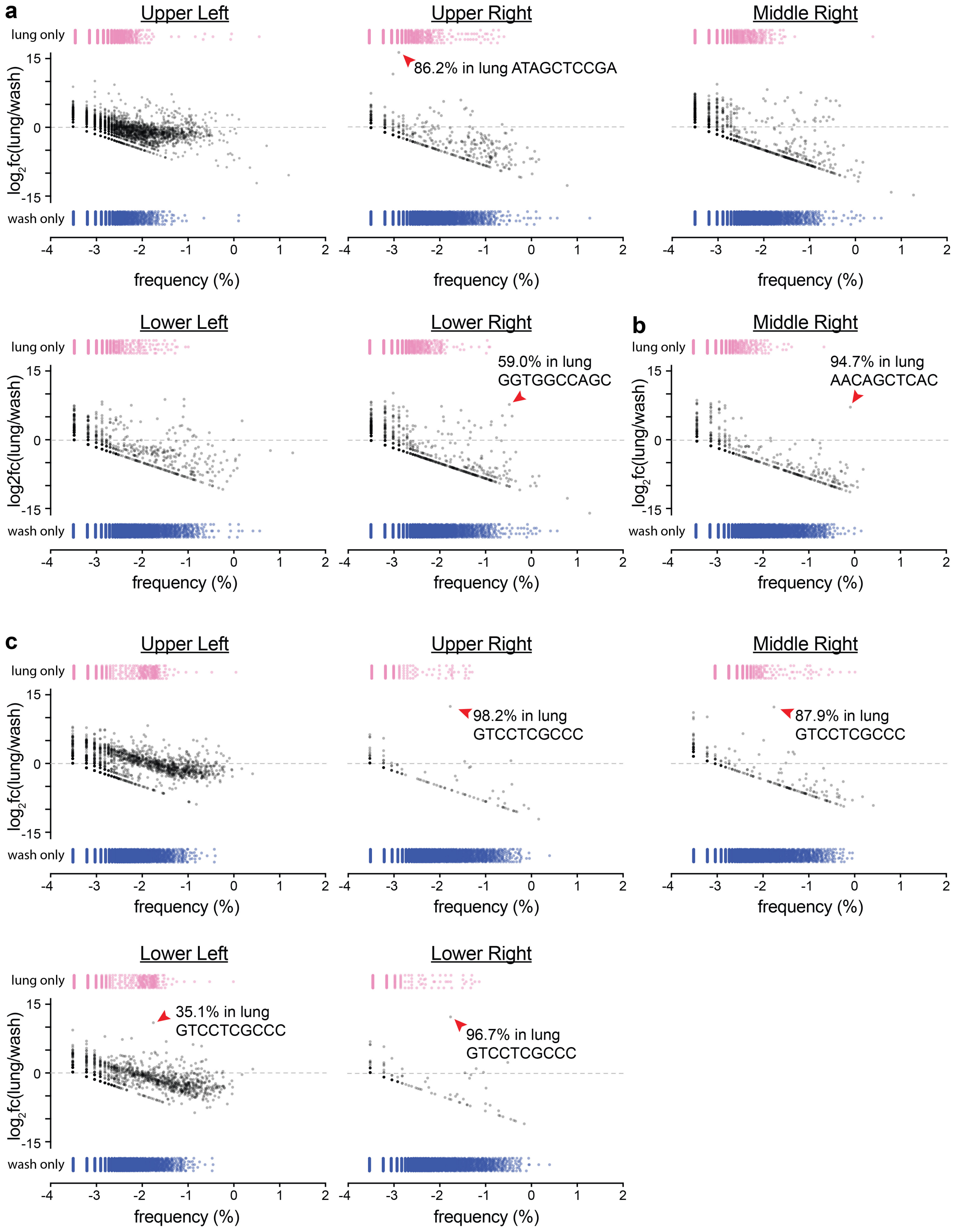

Supplementary Figure 6. Bottlenecks result in stochastic outgrowth of viruses in lung lobes. Associated with Figures 6-7. HA barcode frequencies were determined in the nasal wash (3 dpi) and lung lobes (5 dpi) for a) ferret 34, b) ferret 35, and c) ferret 36. The log2-fold change (log_2fc) in frequency between the lung and the nasal wash was plotted as a function of barcode frequency in the nasal wash (black dots). Barcodes present only in the nasal wash (blue) or lung lobe (pink) are plotted as a function of their frequency within their respective populations. Red arrowheads highlight dominant barcodes with frequency > 30%.

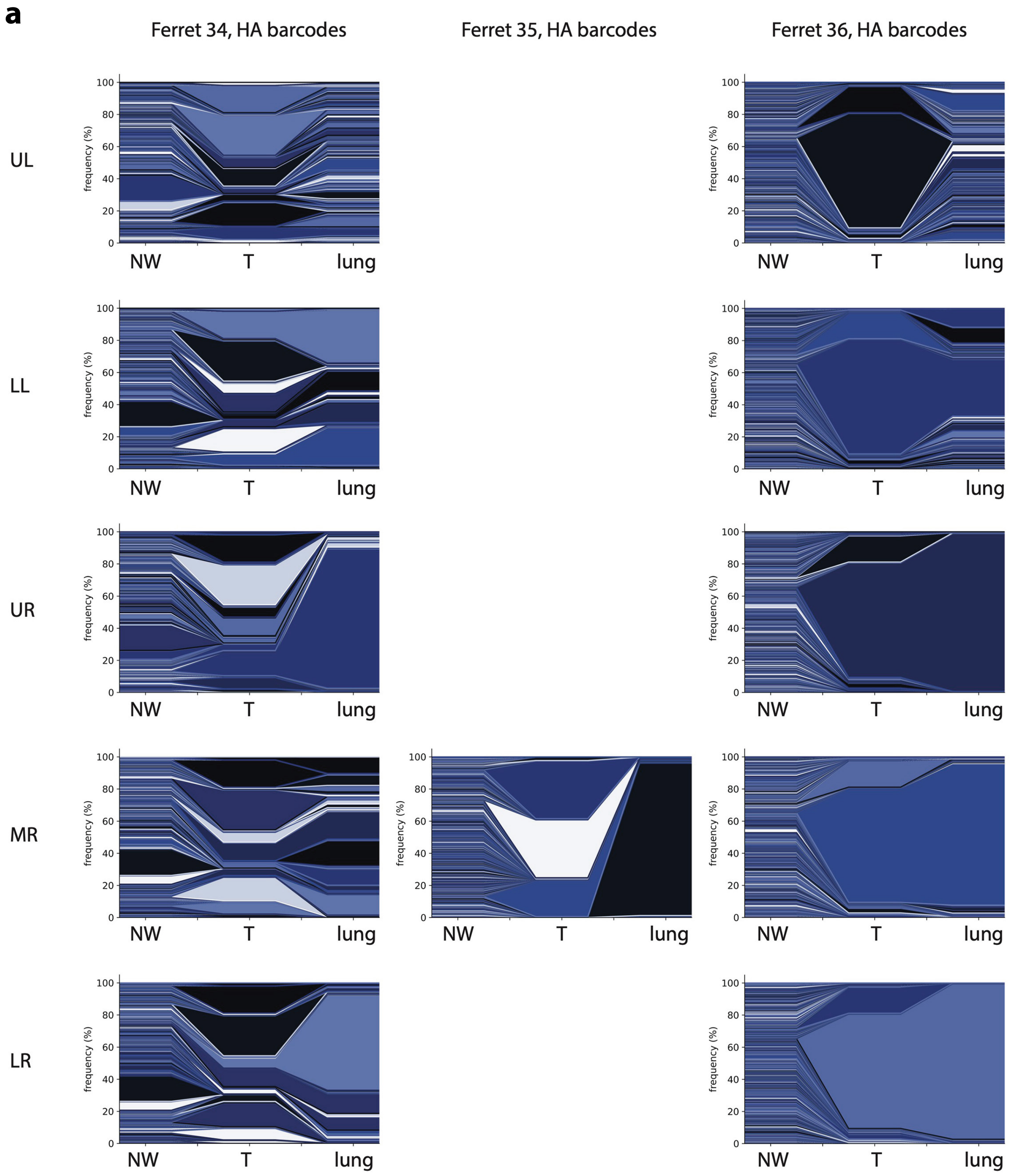

Supplementary Figure 7. Tracking barcodes throughout the respiratory tract. Associated with Figures 5-7. Plots are shown for all lung lobes in which infectious virus was detected. Barcode frequency was plotted to illustrate migration from the upper respiratory tract into distinct lung lobes for (a) HA and (b, next page) PA. Each color represents a unique barcode. Colors are not conserved across samples. NW = nasal wash, 3 dpi. T = trachea. Lung = lung lobe indicated by row.

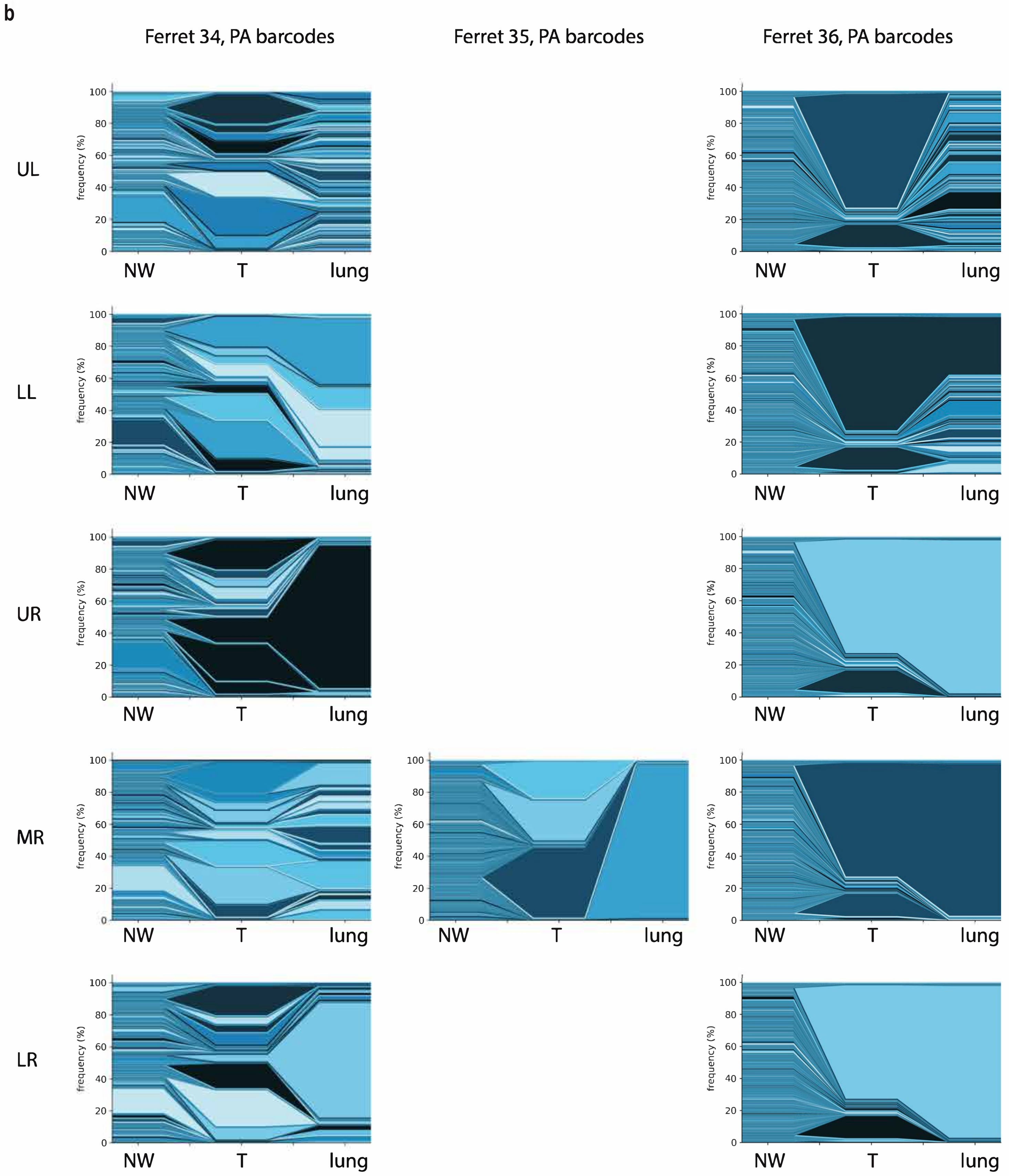
b
Ferret 34, PA barcodes
Ferret 35, PA barcodes
Ferret 36, PA barcodes
UL
LL
UR
MR
LR
frequency (%)
0
20
40
60
80
100
NW
T
lung

	Primer ID	Sequence (5′ - 3′)	Orientation	Purpose	Annealing temperatures
Barcode incorporation/amplification	QuickLib_PASTN_10mer-B	GCGGCTGTGCGAACGCATTCTGGCGTAGGNN NNNNNNNNTGCTTAATGCATCTTGGTTCAACTC CTTCCTC	Forward	template	69ºC
	barcode_amp_FOR	CTACTGCGTCTCAGACCGGCTGGCGGCTGTG CGAACGCATTC	Forward	PA barcode amplification	69ºC
	barcode_amp_REV	AGCTACCGTCTCAGTGCATGTGTGAGGAAGGA GTTGAACCAAGATGC	Reverse	PA barcode amplification	
	mut HA-K153E FOR	GAAAAAGGAAATTCATACCCAAAGCTC	Forward	iPCR	62ºC
	mut HA-K153E REV v2	AACTAGCCATATTAAATTTTTGTAGAAGC	Reverse	iPCR	
	NheI barcode template	TTCTGGATGTGCAGCAACGGCAGCCTCCAGTG TAGGATTTGCATTTAAGCTAGCNNNNNNNNNNT GGTACTGGTAGTCTCCCTGG	Forward	template	54ºC
	PstI barcode template	TTCTGGATGTGCAGCAACGGCAGCCTCCAGTG TAGGATTTGCATTTAACTGCAGNNNNNNNNNNT GGTACTGGTAGTCTCCCTGG	Forward	template	54ºC
	bc_amp_FOR_v2	TCCACGTCTCACTGGGAGCCATCAGCTTCTGG ATGTGCAGC	Forward	HA barcode amplification	54ºC
	bc_amp_REV_v2	CTAGCGCTAGCGTCTCGAGACTACCAGTACC	Reverse	HA barcode amplification	
Reverse Transcriptase	uni12_primer	AGCAAAAGCAGG		Primers for cDNA synthesis of whole gene segments	
	PA_amplicon	GCACTATGGCACACTGGTAATC		Primers for cDNA synthesis of barcoded amplicons	
	HA_amplicon	TGGGGTAAAGCTGGAATCAA		Primers for cDNA synthesis of barcoded amplicons	
H1N1 PCR amplification primers for WGS	PB2_CA04_F	ATA TAC GCG TAG CGA AAG CAG GTC AA	Forward	Primers for the H1N1 CA04 PB2 segment**	67ºC
	PB2_CA04_R	ATA TAC GCG TAG TAG AAA CAA GGT CG	Reverse		
	PB1_CA04_F	ATA TAC GCG TAG CGA AAG CAG GCA AA	Forward	Primers for the H1N1 CA04 PB1 segment	67ºC
	PB1_CA04_R	ATA TAC GCG TAG TAG AAA CAA GGC AT	Reverse		
	PA_CA04_F	ATA TAC GCG TAG CGA AAG CAG GTA CT	Forward	Primers for the H1N1 CA04 PA segment	63ºC
	PA_CA04_R	ATA TAC GCG TAG TAG AAA CAA GGT AC	Reverse		
	HA_CA04_F-v2	AG CAA AAG CAG GGG AAA ACA A	Forward	Primers for the H1N1 CA04 HA segment	61ºC
	HA_CA04_R-v2	AG TAG AAA CAA GGG TGT TTT TC	Reverse		
	NP_CA04_F	ATA TAC GCG TAG CAA AAG CAG GGT AG	Forward	Primers for the H1N1 CA04 NP segment	65ºC
	NP_CA04_R	ATA TAC GCG TAG TAG AAA CAA GGG TA	Reverse		
	NA_CA04_F	ATA TAC GCG TAG CAA AAG CAG GAT TT	Forward	Primers for the H1N1 CA04 NA segment	64ºC
	NA_CA04_R	ATA TAC GCG TAG TAG AAA CAA GGA GT	Reverse		
	M_CA04_F	ATA TAC GCG TAG CAA AAG CAG GTA GA	Forward	Primers for the H1N1 CA04 M segment	63ºC
	M_CA04_R	ATA TAC GCG TAG TAG AAA CAA GGT AG	Reverse		
	NS_CA04_F	ATA TAG GCG TAG CAA AAG CAG GGT GA	Forward	Primers for the H1N1 CA04 NS segment	67ºC
	NS_CA04_R	ATA TAC GCG TAG TAG AAA CAA GGG TG	Reverse		
H1N1 PCR amplification primers for amplicon sequencing	PA_noUMI_fwd	GGA GTT CAG ACG TGT GCT CTT CCG ATC TGC ACT ATG GCA CAC TGG TAA TC	Forward	Primers for the amplification of PA amplicons	63ºC
	PA_rev	TCT TTC CCT ACA CGA CGC TCT TCC GAT CTG CAT GTG TGA GGA AGG AG	Reverse		
	HA_noUMI_fwd	GGA GTT CAG ACG TGT GCT CTT CCG ATC TTG GGG TAA AGC TGG AAT CAA	Forward	Primers for the amplification of HA amplicons	59ºC
	HA_rev	TCT TTC CCT ACA CGA CGC TCT TCC GAT CCA CTG TAG AGA CCC ATT AGA	Reverse		
Universal adaptors for Illumina sequencing	uni_fwd_adaptors	CAA GCA GAA GAC GGC ATA CGA GAT **NNN NNN** GTG ACT GGA GTT CAG ACG TGT GCT CTT *	Forward	Primers for the amplification of PA amplicons	72ºC
	uni_rev_adaptors	AAT GAT ACG GCG ACC ACC GAG ATC TAC ACT CTT TCC CTA CAC GAC GCT CTT CC	Reverse		
**Supplementary Table 1.** List of primers used for design and deep sequencing of barcoded-H1N1 virus. * Indexes for "NNN NNN" sequence are listed in Table 2.					
** CA04 primers are compatible with CA07					

	Index Number	Sequence in adapter
Indexes used for illumina sequencing (MiSeq - 250v2)	A0001	ATC ACG
	A0002	CGA TGT
	A0003	TTA GGC
	A0004	TGA CCA
	A0005	ACA GTG
	A0006	GCC AAT
	A0007	CAG ATC
	A0008	ACT TGA
	A0009	GAT CAG
	A0010	TAG CTT
	A0011	GGC TAC
	A0012	CTT GTA
	A0013	AGT CAA
	A0014	AGT TCC
	A0015	ATG TCA
	A0016	CCG TCC
	A0018	GTC CGC
	A0019	GTG AAA
	A0020	GTG GCC
	A0021	GTT TCG
	A0022	CGT ACG
	A0023	GAG TGG
	A0025	ACT GAT
	A0027	ATT CCT
Nextera i5 indexes used for illumina sequencing (MiSeq - 300v3)	502	CTCTCTAT
	503	TATCCTCT
	505	GTAAGGAG
	506	ACTGCATA
	507	AAGGAGTA
	508	CTAAGCCT
Nextera i7 indexes used for illumina sequencing (MiSeq - 300v3)	701	TCGCCTTA
	702	CTAGTACG
	703	TTCTGCCT
	704	GCTCAGGA
	705	AGGAGTCC
	706	CATGCCTA
	707	GTAGAGAG
	710	CAGCCTCG
	711	TGCCTCTT
	712	TCCTCTAC
	714	TCATGAGC
	715	CCTGAGAT

Supplementary Table 2. List of indexes used for deep sequencing of barcoded-H1N1 virus

bioproject_accessi	sample_name	BioSample_access	SRA_accession	title
PRJNA746307	NheI_skewed_amplified_IAV	SAMN20200962	SRR15168778	barcoded Influenza A virus - NheI skewed amplified library
PRJNA746307	NheI_skewed_plasmid_IAV	SAMN20201485	SRR15168777	barcoded Influenza A virus - NheI skewed plasmid library
PRJNA746307	NheI_skewed_rescue_IAV	SAMN20201486	SRR15168776	barcoded Influenza A virus - NheI skewed rescue library
PRJNA746307	PstI_skewed_amplified_IAV	SAMN20201487	SRR15168775	barcoded Influenza A virus - PstI skewed amplified library
PRJNA746307	PstI_skewed_plasmid_IAV	SAMN20201488	SRR15168774	barcoded Influenza A virus - PstI skewed plasmid library
PRJNA746307	PstI_skewed_rescue_IAV	SAMN20201489	SRR15168773	barcoded Influenza A virus - PstI skewed rescue library
PRJNA746317	NheI_K153E_amplified_IAV	SAMN20201490	SRR15175246	barcoded Influenza A virus - NheI K153E amplified library
PRJNA746317	NheI_K153E_plasmid_IAV	SAMN20201491	SRR15175245	barcoded Influenza A virus - NheI K153E plasmid library
PRJNA746317	NheI_K153E_rescue_IAV	SAMN20201492	SRR15175244	barcoded Influenza A virus - NheI K153E rescue library
PRJNA746317	PstI_K153E_amplified_IAV	SAMN20201493	SRR15175243	barcoded Influenza A virus - PstI K153E amplified library
PRJNA746317	PstI_K153E_plasmid_IAV	SAMN20201494	SRR15175242	barcoded Influenza A virus - PstI K153E plasmid library
PRJNA746317	PstI_v1_K153E_rescue_IAV	SAMN20201495	SRR15175241	barcoded Influenza A virus - PstI K153E rescue library v1
PRJNA746317	PstI_v2_K153E_rescue_IAV	SAMN20201496	SRR15175240	barcoded Influenza A virus - PstI K153E rescue library v2
PRJNA746319	NheI_stock_virus_K153E	SAMN20247425	SRR15176827	barcoded Influenza A virus - NheI K153E stock for in vivo infections
PRJNA746319	PstI_stock_virus_K153E	SAMN20247426	SRR15176826	barcoded Influenza A virus - PstI K153E stock for in vivo infections
PRJNA746319	ferret_34_nasal_wash_1dpi	SAMN20251314	SRR15176815	barcoded Influenza A virus - Nasal wash from ferret 34 at 1dpi with NheI
PRJNA746319	ferret_34_nasal_wash_3dpi	SAMN20251315	SRR15176804	barcoded Influenza A virus - Nasal wash from ferret 34 at 3dpi with NheI
PRJNA746319	ferret_34_nasal_wash_5dpi	SAMN20251316	SRR15176803	barcoded Influenza A virus - Nasal wash from ferret 34 at 5dpi with NheI
PRJNA746319	ferret_35_nasal_wash_1dpi	SAMN20251317	SRR15176802	barcoded Influenza A virus - Nasal wash from ferret 35 at 1dpi with NheI
PRJNA746319	ferret_35_nasal_wash_3dpi	SAMN20251318	SRR15176801	barcoded Influenza A virus - Nasal wash from ferret 35 at 3dpi with NheI
PRJNA746319	ferret_35_nasal_wash_5dpi	SAMN20251319	SRR15176800	barcoded Influenza A virus - Nasal wash from ferret 35 at 5dpi with NheI
PRJNA746319	ferret_36_nasal_wash_1dpi	SAMN20251320	SRR15176799	barcoded Influenza A virus - Nasal wash from ferret 36 at 1dpi with NheI
PRJNA746319	ferret_36_nasal_wash_3dpi	SAMN20251321	SRR15176798	barcoded Influenza A virus - Nasal wash from ferret 36 at 3dpi with NheI
PRJNA746319	ferret_36_nasal_wash_5dpi	SAMN20251322	SRR15176825	barcoded Influenza A virus - Nasal wash from ferret 36 at 5dpi with NheI
PRJNA746319	ferret_34_trachea	SAMN20251323	SRR15176824	barcoded Influenza A virus - trachea from ferret 34 infected with NheI
PRJNA746319	ferret_35_trachea	SAMN20251324	SRR15176823	barcoded Influenza A virus - trachea from ferret 35 infected with NheI
PRJNA746319	ferret_36_trachea	SAMN20251325	SRR15176822	barcoded Influenza A virus - trachea from ferret 36 infected with NheI
PRJNA746319	ferret_34_lung_lobes	SAMN20251326	SRR15176821	barcoded Influenza A virus - lung lobes from ferret 34 infected with NheI
PRJNA746319	ferret_35_lung_lobes	SAMN20251327	SRR15176820	barcoded Influenza A virus - lung lobes from ferret 35 infected with NheI
PRJNA746319	ferret_36_lung_lobes	SAMN20251328	SRR15176819	barcoded Influenza A virus - lung lobes from ferret 36 infected with NheI
PRJNA746319	mouse_Nhe_L_lung	SAMN20251329	SRR15176817	barcoded Influenza A virus - lung homogenate from mouse 4 infected with NheI
PRJNA746319	mouse_Nhe_LL_lung	SAMN20251330	SRR15176818	barcoded Influenza A virus - lung homogenate from mouse 5 infected with NheI
PRJNA746319	mouse_Nhe_LR_lung	SAMN20251331	SRR15176816	barcoded Influenza A virus - lung homogenate from mouse 6 infected with NheI
PRJNA746319	mouse_Nhe_NA_lung	SAMN20251332	SRR15176814	barcoded Influenza A virus - lung homogenate from mouse 3 infected with NheI
PRJNA746319	mouse_Nhe_R_lung	SAMN20251333	SRR15176813	barcoded Influenza A virus - lung homogenate from mouse 1 infected with NheI
PRJNA746319	mouse_Nhe_RR_lung	SAMN20251334	SRR15176812	barcoded Influenza A virus - lung homogenate from mouse 2 infected with NheI
PRJNA746319	mouse_Pst_L_lung	SAMN20251335	SRR15176811	barcoded Influenza A virus - lung homogenate from mouse 7 infected with NheI
PRJNA746319	mouse_Pst_LL_lung	SAMN20251336	SRR15176810	barcoded Influenza A virus - lung homogenate from mouse 8 infected with NheI
PRJNA746319	mouse_Pst_LR_lung	SAMN20251337	SRR15176809	barcoded Influenza A virus - lung homogenate from mouse 9 infected with NheI
PRJNA746319	mouse_Pst_NA_lung	SAMN20251338	SRR15176808	barcoded Influenza A virus - lung homogenate from mouse 12 infected with NheI
PRJNA746319	mouse_Pst_R_lung	SAMN20251339	SRR15176807	barcoded Influenza A virus - lung homogenate from mouse 10 infected with NheI
PRJNA746319	mouse_Pst_RR_lung	SAMN20251340	SRR15176806	barcoded Influenza A virus - lung homogenate from mouse 11 infected with NheI
PRJNA746319	negative_controls	SAMN20251341	SRR15176805	barcoded Influenza A virus - negative controls used throughout deep sequencing experiments
PRJNA746307	amplified_virus_CA07bc_Nhe_skewed_whole_genome	SAMN20284438	SRR15177608	barcoded Influenza A virus - whole genome sequencing of NheI skewed amplified library
PRJNA746307	amplified_virus_CA07bc_Pst_skewed_whole_genome	SAMN20284439	SRR15177607	barcoded Influenza A virus - whole genome sequencing of PstI skewed amplified library
PRJNA746317	amplified_virus_CA07bc_Nhe_K153E_whole_genome	SAMN20284440	SRR15178010	barcoded Influenza A virus - whole genome sequencing of NheI K153E amplified library
PRJNA746317	amplified_virus_CA07bc_Pst_K153E_whole_genome	SAMN20284441	SRR15178009	barcoded Influenza A virus - whole genome sequencing of PstI K153E amplified library
PRJNA746319	complete_HA_for_ferret_34_lung	SAMN20284442	SRR16027395	barcoded Influenza A virus - complete HA sequencing of lung lobes from ferret 34
PRJNA746319	complete_HA_for_ferret_35_lung	SAMN20284443	SRR16027394	barcoded Influenza A virus - complete HA sequencing of lung lobes from ferret 35
PRJNA746319	complete_HA_for_ferret_36_lung	SAMN20284444	SRR16027393	barcoded Influenza A virus - complete HA sequencing of lung lobes from ferret 36
PRJNA746319	negative_controls_WGS	SAMN20284445	SRR15176805	Negative controls used for all WGS of barcoded flu sequencing

Supplementary Table 3: Accession numbers for sequencing data
