# Supplementary material for: Influenza A virus undergoes compartmentalized replication in vivo dominated by stochastic bottlenecks
Source: Nat Commun. 2022 Jun 14;13:3416. doi: 10.1038/s41467-022-31147-0 (PMC9197827; doi:10.1038/s41467-022-31147-0)
Supplement: Supplementary file 3 — Reporting Summary [file 41467_2022_31147_MOESM3_ESM.pdf]

nature portfolio

Corresponding author(s): Andrew Mehle

Last updated by author(s): 5/25/2022

Reporting Summary

Nature Portfolio wishes to improve the reproducibility of the work that we publish. This form provides structure for consistency and transparency in reporting. For further information on Nature Portfolio policies, see our Editorial Policies and the Editorial Policy Checklist.

Statistics

For all statistical analyses, confirm that the following items are present in the figure legend, table legend, main text, or Methods section.

n/a	Confirmed	
☐	☒	The exact sample size (*n*) for each experimental group/condition, given as a discrete number and unit of measurement
☐	☒	A statement on whether measurements were taken from distinct samples or whether the same sample was measured repeatedly
☐	☒	The statistical test(s) used AND whether they are one- or two-sided<br>*Only common tests should be described solely by name; describe more complex techniques in the Methods section.*
☒	☐	A description of all covariates tested
☐	☒	A description of any assumptions or corrections, such as tests of normality and adjustment for multiple comparisons
☐	☒	A full description of the statistical parameters including central tendency (e.g. means) or other basic estimates (e.g. regression coefficient) AND variation (e.g. standard deviation) or associated estimates of uncertainty (e.g. confidence intervals)
☐	☒	For null hypothesis testing, the test statistic (e.g. *F*, *t*, *r*) with confidence intervals, effect sizes, degrees of freedom and *P* value noted<br>*Give P values as exact values whenever suitable.*
☒	☐	For Bayesian analysis, information on the choice of priors and Markov chain Monte Carlo settings
☒	☐	For hierarchical and complex designs, identification of the appropriate level for tests and full reporting of outcomes
☐	☒	Estimates of effect sizes (e.g. Cohen's *d*, Pearson's *r*), indicating how they were calculated

Our web collection on statistics for biologists contains articles on many of the points above.

Software and code

Policy information about availability of computer code

Data collection	Sequencing data were generated using the Illumina MiSeq system or Oxford Nanopore Technologies GridION machine with bases called in real time using the ONT software package Guppy 3.2.6.

Data analysis	Detailed protocols and code available at https://github.com/mehlelab/barcoded_flu_analysis.<br><br>For Illumina sequencing of barcodes, raw FASTQ paired reads were demultiplexed, merged and aligned using BBMap Tools v38.87. Reads with lengths the same as the average insert size were aligned, sorted and indexed with Samtools (v1.11, htslib v1.11). BAM files with aligned reads were processed and trimmed using command line tools (Seqtk v1.3, Bash v3.2.57). UMI-tools (v1.1.1) was used for clustering via the adjacency network-based clusterer method. Cluster frequencies were generated and visualized via a custom Python pipeline (Python v3.8.5, Pandas v1.1.3, Matplotlib v3.3.2, Numpy v1.19.2) or in Prism 9.<br><br>For Illumina sequencing of full HA, FASTQ paired reads were demultiplexed and merged using bbmerge (BBMap Tools v38.87) and mapped using a Burrows-Wheeler alignment (BWA v.0.7.17). Using Samtools (v1.11, htslib v1.11), we sorted aligned reads and called variants using LoFreq (v2.1.5). SNVs were annotated using SnpEff (v5.0e) and the resulting variant call format (VCF) files were manipulated using bcftools (v1.11). Plots were generated using custom bioinformatic pipelines in Python language (Python v3.8.5, Pandas v1.1.3, Matplotlib v3.3.2, Seabornv0.11.2, Numpy v1.19.2).<br><br>For ONT data, Minimap2 was used to map reads. The Sam2Tsv module (v1.0) from Jvarkit was used to convert the sam to a tsv file for manipulation in Pandas (v1.1.3). UMI-tools (v1.1.1) was used to generate consensus barcode clusters via directional network-based clusterer. Results were visualized with Prism 9.<br><br>For rarefaction-extrapolation, iNEXT (v2.0.20) was implemented in R (v4.0.2).

For manuscripts utilizing custom algorithms or software that are central to the research but not yet described in published literature, software must be made available to editors and reviewers. We strongly encourage code deposition in a community repository (e.g. GitHub). See the Nature Portfolio guidelines for submitting code & software for further information.

Data

Policy information about availability of data

All manuscripts must include a data availability statement. This statement should provide the following information, where applicable:

- Accession codes, unique identifiers, or web links for publicly available datasets
- A description of any restrictions on data availability
- For clinical datasets or third party data, please ensure that the statement adheres to our policy

All sequencing files have been deposited as BioProjects PRJNA746307, PRJNA746319, and PRJNA746317 with details and SRA accessions in Supplementary Table 3. Source data are provided with this paper.

Field-specific reporting

Please select the one below that is the best fit for your research. If you are not sure, read the appropriate sections before making your selection.

☒ Life sciences ☐ Behavioural & social sciences ☐ Ecological, evolutionary & environmental sciences

For a reference copy of the document with all sections, see nature.com/documents/nr-reporting-summary-flat.pdf

Life sciences study design

All studies must disclose on these points even when the disclosure is negative.

Sample size	Sample sizes were determined following best practices in the field (e.g. Matsuoka , et al. 2009. Curr Protoc Microbiol. 13:15G.2.1-15G.2.29. PMID: 19412910; Matsuoka , et al. 2009. Curr Protoc Microbiol. 13:15G.3.1-15G.3.30. PMID: 19412911), and the authors' prior experience. This includes large enough samples within a technical replicate and sufficient number of independent biological replications to provide the opportunity for representative results, while balancing feasibility for animal experiments.
Data exclusions	No data were excluded from analyses.
Replication	All data represent successfully replicated experiments containing at least n=3 biological replicates. For tissue culture studies, each biological replicate also contained at least 3 technical replicates. All raw sequencing data have been deposited as described above so analyses can be replicated by other groups.
Randomization	Mice were randomly assigned to infection groups. Ferrets were not randomized as only one infection condition was used.
Blinding	Blinding was not performed.

Reporting for specific materials, systems and methods

We require information from authors about some types of materials, experimental systems and methods used in many studies. Here, indicate whether each material, system or method listed is relevant to your study. If you are not sure if a list item applies to your research, read the appropriate section before selecting a response.

Materials & experimental systems

n/a	Involved in the study
[x]	[ ] Antibodies
[ ]	[x] Eukaryotic cell lines
[x]	[ ] Palaeontology and archaeology
[ ]	[x] Animals and other organisms
[x]	[ ] Human research participants
[x]	[ ] Clinical data
[x]	[ ] Dual use research of concern

Methods

n/a	Involved in the study
[x]	[ ] ChIP-seq
[x]	[ ] Flow cytometry
[x]	[ ] MRI-based neuroimaging

Eukaryotic cell lines

Policy information about cell lines

Cell line source(s)	Madin-Darby canine kidney (MDCK) ATCC CCL-22<br>human embryonic kidney cells (HEK293T) ATCC CRL-3216<br>MDCK-SIAT1-TMPRSS2 cells (gift from Jesse Bloom)
Authentication	Cells were verified by ATCC using STR.
Mycoplasma contamination	Routine mycoplasma testing is performed on all cells (Lonza MycoAlert) and cells tested negative.
Commonly misidentified lines<br>(See ICLAC register)	not applicable

Animals and other organisms

Policy information about studies involving animals; ARRIVE guidelines recommended for reporting animal research

Laboratory animals	9-week old female BALB/c mice (Charles River Labs) housed at 22C, ~30% relative humidity with a 12hr light-dark cycle.<br>12-week old male ferrets (Triple F Farms, Sayre, PA)
Wild animals	not used in this study
Field-collected samples	not used in this study
Ethics oversight	All mouse experiments were approved by the University of Wisconsin Madison Institutional Animal Care and Use Committee.<br>All ferret experiments were approved by the St Jude Children's Research Hospital Animal Care and Use Committee.

Note that full information on the approval of the study protocol must also be provided in the manuscript.
